# Linearizing and Forecasting: A Reservoir Computing Route to Digital Twins of the Brain

**DOI:** 10.1002/advs.202517234

**Published:** 2026-03-19

**Authors:** Gabriele Di Antonio, Tommaso Gili, Andrea Gabrielli, Maurizio Mattia

**Affiliations:** ^1^ “Enrico Fermi” Research Center ‐ CREF Rome Italy; ^2^ Natl. Center for Radiation Protection and Computational Physics Istituto Superiore di Sanità Rome Italy; ^3^ Networks Unit IMT Scuola Alti Studi Lucca Lucca Italy; ^4^ Dip. di Ingegneria Civile, Informatica e delle Tecnologie Aeronautiche Università degli Studi “Roma Tre” Rome Italy

**Keywords:** data‐driven digital‐twins, koopman operator, recurrent neural networks, reservoir computing, resting state fmri

## Abstract

Exploring the dynamics of complex systems such as the human brain is challenging due to inherent uncertainties and the limited availability of high‐quality data. Here, we develop a mathematical theory for noisy linear recurrent neural networks (lRNNs) within the reservoir computing framework and demonstrate their effectiveness in constructing autonomous in silico replicas – digital‐twins – of brain activity. We show that the Laplace‐transform poles of high‐dimensional inferred lRNNs directly encode the spectral properties of observed systems and are linked to the kernels of auto‐regressive models. Notably, our approach enables accurate recovery of the system's linear spectrum even when observations undergo conventional preprocessing, including band‐pass filtering pipelines commonly used in neural recordings and resting‐state fMRI. In these regimes, established techniques such as dynamic mode decomposition often produce spurious spectral estimates. Applying our framework to resting‐state fMRI, we successfully predict and decompose BOLD activity into spatiotemporal modes in a low‐dimensional latent state space confined around a single equilibrium point. The inferred lRNNs provide interpretable signatures that differentiate subjects and brain areas, supporting biologically meaningful clustering. This flexible digital‐twin framework opens the door to virtual experiments and computationally efficient real‐time adaptive learning, offering a promising avenue for personalized medicine and intervention strategies.

## Graphical abstract

A new approach uses simple neural networks to create digital twins of brain activity, capturing how different patterns unfold over time. The method generates and recovers key dynamics even from noisy data. When applied to fMRI, it predicts brain signals and reveals distinctive activity patterns across regions and individuals, opening possibilities for virtual tests and personalized diagnostic and interventions.

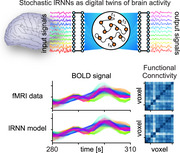

## 1 Introduction

The study of complex dynamical systems has a central role in contemporary scientific research. Over recent decades, a plethora of data‐driven methodologies has emerged, denoting a field in rapid evolution that is capable to addressing systems with increasing complexity. Among these methodologies, machine learning techniques have become increasingly popular due to their effectiveness in modeling complex datasets and delivering accurate predictions [1, 2, 3].

Within the landscape of machine learning methods, reservoir computing (RC) has emerged as a powerful framework for processing temporal data with remarkable computational efficiency. RC leverages high‐dimensional recurrent neural networks (RNNs) with fixed internal couplings to transform input time series into rich, high‐dimensional representations [4, 5]. This transformation enables the prediction of future samples by simply reading out the current state of the network, effectively capturing the system's dynamics [6, 7, 8]. The elegance of this approach lies in conceptualizing the readout as a projection onto a manifold learned through linear regression, echoing the efficient coding strategies observed in biological neural networks [9, 10, 11]. Consequently, RNNs have become invaluable tools in neuroscience, aiding in hypothesis generation and providing analytical frameworks for understanding neural computations [12, 13, 14, 15].

However, as machine learning has become increasingly popular, some clarity of understanding may have been lost in the pursuit of better predictions. While the inferred models are often quite accurate, they can sometimes lack interpretability [16, 17, 18]. A valuable framework to address this limitation is provided by Koopman theory, which offers clear insights into the system dynamics by mapping nonlinear systems into simple yet higher‐dimensional dynamics [19, 20, 21, 22]. This approach involves the development of data‐driven methods aimed at computing finite‐dimensional linear representations of nonlinear systems within a suitable functional space. Once an equivalent linear system is inferred, interpretability becomes straightforward, allowing to gain understanding of the time scales and dynamical modes of the observed systems.

**FIGURE 1:**
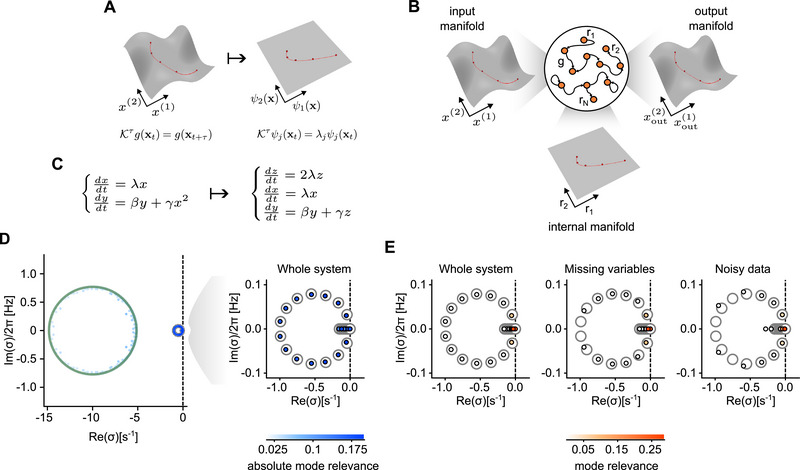
lRNN encodes a linear representation of the input system. (A) Nonlinear dynamics can be projected onto linear manifolds. Within the infinite functional space of the system observables, the eigenfunctions of the Koopman operator establish a basis for decomposing nonlinear dynamics into simpler linear processes. (B) Reservoir computing (RC) with recurrent neural networks (RNNs). After ‘learning’, the readout weights $W^{\mathrm{out}}$ are set to reproduce the system observables provided as input. The resulting network is composed of linear units (lRNN) and its post‐learning autonomous dynamics encapsulates a linear representation of the original system. C) Example of a 2‐D nonlinear system that can be decomposed into a set of three linearly evolving variables. (D) Spectrum of a lRNN with N=500 units trained to mimic a 15‐D version of the system in (C) composed of five linear x variables and ten nonlinear y variables. Green circles, spectrum of the lRNN before training. Gray circles, theoretically‐derived eigenvalues of the 20‐D linearized system to be replicated (see text for details). Small circles, Laplace transform poles from the post‐learning autonomous lRNN replicating the example system. Filling colors code the absolute mode relevance ${\left\langle |\Xi_{jk}|\right\rangle }_{j}$. Right panel, zoom in of the left panel showing the 20 most significant modes. (E) Same spectrum as in (D) with filling colors coding the mode relevance ${\left\langle |\Xi_{jk}v_{k}\left(0\right)|\right\rangle }_{j}$ (left panel, see text for details). Central panel, spectrum of a lRNN trained on incomplete information (i.e., omitting all x and 5 y variables). Right panel, spectrum of a lRNN trained on the same observables but perturbed by noise.

The capability to predict future states of a dynamical system based on past observations allows, in principle, to build a replica of the same system. Indeed, predicted observations can be fed back as input resulting in a generative model. Both Koopman‐based methods (Figure 1A) and RC approaches (Figure 1B) have the potential to generate these digital copies by producing time series statistically equivalent to those generated by the original systems. The inferred generative models can then serve as ‘digital twins’ – a concept that has emerged as a transformative paradigm in the study of dynamical and natural systems [23, 24]. Such digital twins can be effective tools for analyzing and simulating their physical counterparts. Successful examples include digital copies of brain activity designed to assist neurosurgeons in dealing with drug‐resistant epileptic patients [25, 26, 27]. Furthermore, these tools can provide a complementary workbench for designing stimulation approaches to be tested in silico, ultimately leading to control systems for neurorehabilitation [28].

In this study, we investigate the potential of RNNs with linear units to implement effective digital twins of single‐subject brain dynamics, measured as the vascular response to neuronal activity (i.e., the BOLD signal) extracted from functional magnetic resonance imaging (fMRI). We demonstrate the autoregressive nature of this approach and its effectiveness in representing whole‐brain activity in a low‐dimensional latent state space. Such a reduced representational complexity mitigates the risk of overfitting often associated with one‐to‐one fitted physical models, especially in realistic scenarios where only a limited number of observations are available and only a partial view of the system can be experimentally accessed. The integration of endogenous memoryless fluctuations is another essential component that enables the digital twin to self‐sustain a noisy dynamics statistically equivalent to the one experimentally measured and, crucially, to recover the correct spectral content from filtered observations.

## 2 Results

### 2.1 Linear RNNs can Copy Nonlinear Systems

We start investigating the reservoir computing abilities of a RNN with N linear units (lRNN). The state of the lRNN is the vector r(t) whose elements are the activities $r_{i}\left(t\right)$ of each unit, following the linear dynamics

(1) $$\tau \dot{r}\left(t\right)=Wr\left(t\right)+W^{\mathrm{in}}\omega \left(t\right)-r\left(t\right)\;$$

The synaptic matrix $W\in R^{N\times N}$ determines the recurrent input Wr, which, together with the contributions from the external environment $W^{\mathrm{in}}\omega \left(t\right)$, composes the total input h that each unit receives. In general, these currents are nonlinearly transformed by an input–output gain function Φ(h) we assume here to be Φ(h)=h – a condition usually associated with unit activities perturbatively fluctuating around the quiescent state (see Methods Section). Network units receive a source of external input due to the M observables $\omega_{i}\left(t\right)$ of the systems under investigation, mediated by the synaptic matrix $W^{\mathrm{in}}\in R^{N\times M}$. From Equation (1) the network state at any time t results then to be

(2) $$r\left(t\right)=\int_{-\infty }^{t}e^{\left(W-I\right)\left(t-t^{\prime }\right)/\tau }W^{\mathrm{in}}\omega \left(t^{\prime }\right)dt^{\prime }\;$$

where $I\in R^{N\times N}$ is the identity matrix.

If the lRNN is capable to display the so‐called ‘generalized synchronization’ property in response to the input $W^{\mathrm{in}}\omega$ [29, 30], r(t) effectively embeds the dynamics of the ‘whole’ observed system into its state space. This because the lRNN incorporates the ‘echo’ [4, 31, 32] of past observations implementing a dimensional embedding *a la* Takens [33, 34, 35]. In this RC framework, for sufficiently high N a simple linear transformation capable to approximately map the network state to a target output exists

(3) $$\omega \left(t\right)\approx W^{\mathrm{out}}r\left(t\right)=\int_{0}^{\infty }G\left(s\right)\omega \left(t-s\right)ds$$

with synaptic weights $W^{\mathrm{out}}\in R^{M\times N}$ computed via a simple linear regression [4, 5]. The linear kernels $G\left(s\right)=W^{\mathrm{out}}E^{(W-I)(s)/\tau }W^{\mathrm{in}}\in R^{M\times M}$ (i.e., the Green functions) resulting from Equation (2) reveals the autoregressive nature of the RC approach in this lRNN case.

This concept can be better clarified by resorting to the eigendecomposition of the synaptic matrix $W=Q\Lambda Q^{-1}$. Here, Λ is the diagonal matrix of the eigenvalues ($\Lambda_{ii}=\lambda_{i}$) and the eigenvectors are the columns of Q: $W|Q_{i}\rangle =\lambda_{i}|Q_{i}\rangle$. By applying this decomposition to the kernel expression, a superposition of N exponential modes results:

(4) $$G\left(s\right)=\sum_{n=1}^{N}J_{n}e^{(\lambda_{n}-1)s/\tau }\;$$

where $J_{n}=W^{\mathrm{out}}|Q_{n}\rangle \left\langle Q_{n}^{-1}\right|W^{\mathrm{in}}$ are N rank‐1 matrices with $\left\langle Q_{n}^{-1}\right|$ being the n‐th row of the inverse matrix $Q^{-1}$ (see Methods Section). The convolution in Equation (3) is integrable if the spectral radius $\rho =\max_{n\in [1,N]}\text{Re}\;\lambda_{n}$ of W is smaller than 1. Under this condition, unperturbed lRNNs (ω=0) have a stable equilibrium point at r=0, and self‐consistency equation Equation (3) describes a continuous‐time autoregressive (AR) model with order N (number of exponential modes in G(s)). This relationship between RC with lRNNs and AR models generalizes previous results obtained in the discrete‐time domain [36, 37]. As we will see later, the order of the equivalent AR model can be much smaller than N as many $J_{n}$ are usually close to 0.

By replacing the observables ω(t) provided as input with the reconstructed once from Equation (3), the lRNN results to follow the autonomous dynamics

(5) $$\tau \dot{r}=\tilde{W}r-r$$

where $\tilde{W}=W+W^{\mathrm{in}}W^{\mathrm{out}}$ is the updated (i.e., ‘learned') synaptic matrix. If the network can correctly predict the future of the target output, Equation (5) provides a linear representation of the system under analysis. Relying as above on the eigenmode decomposition $\tilde{W}=\tilde{Q}\tilde{\Lambda }{\tilde{Q}}^{-1}$, the projections $v\left(t\right)\equiv {\tilde{Q}}^{-1}r\left(t\right)$ evolve in time as a set of decoupled variables:

(6) $$v_{k}\left(t\right)=v_{k}\left(0\right)e^{({\tilde{\lambda }}_{k}-1)t/\tau }\;\;\;\forall k\in \left[1,N\right]$$

This last step makes it apparent the relationship with the Koopman‐operator theory, as a suited linear combination of these modes eventually implements an effective decomposition of the time series ω(t):

(7) $$\omega \left(t\right)=W^{\mathrm{out}}r\left(t\right)\equiv \Xi v\left(t\right)\;$$

with $\Xi =W^{\mathrm{out}}\tilde{Q}$. The Laplace transform of the reconstructed observable will then have N poles $\sigma_{k}=\left({\tilde{\lambda }}_{k}-1\right)/\tau$ with their related residues, allowing us to represent the dynamical properties of the ‘digital twin’ (i.e., the autonomous lRNN), and linking it to AR models and finite approximations of the Koopman operator (see Methods Section). Indeed, the k‐th residue is proportional to $\Xi_{jk}v_{k}\left(0\right)$ and provides in absolute value the relevance of the k‐th mode in describing the observable $\omega_{j}$, given the initial network state r(0).

As a testing ground for the theoretical framework derived above we consider the following nonlinear system:

(8) $$\left\{\begin{array}{c} \dot{x}=C_{x}x\\ \dot{y}=C_{y}y+C_{yx}x⊙x \end{array}\right.$$

Choosing $C_{x}$ as a diagonal matrix, this system can be mapped onto a linear one by introducing a set of new observables z=x⊙x=diag(x)x (Figure 1C). In this case, the related infinitesimal generator of the Koopman operators is equivalent to the finite matrix K with diagonal blocks $C_{x}$, $2C_{x}$, $C_{y}$.

We trained a lRNN to replicate the relaxation dynamics of this system from the observables ω=[x;y] with $x\in R^{5}$ and $y\in R^{10}$, starting from a random initial condition ω(0). As initial synaptic matrix W we chosen the one associated to ring‐like connectivity (i.e., $W_{i,i+1}=W_{N,1}=\rho$ are the only non‐zero elements) known to have optimal performances in the case of linear units [38]. In Figure 1D, the poles $\sigma_{k}$ of the reconstructed observables from Equation (7) are shown together with the related mode relevance (color intensity). Despite the nonlinearity of the system to replicate, the overlap between the poles $\sigma_{k}$ and the theoretical spectrum of K is remarkable. Furthermore, only 20 out of the N=500 modes have $\left\langle \right|\Xi_{jk}{\left|\right\rangle }_{j}$ significantly different from zero (blue‐colored circles).

Decomposition modes of the trained and autonomous lRNN exhibit damped oscillations following Equation (6) for each pole $\sigma_{k}\in C$. Each mode has an amplitude envelope eRe(σk)t and oscillates at angular frequency Im(σk). Consequently, most modes decay rapidly and have little influence on the dynamics, whereas only a few slow modes, i.e. those with the largest (least negative) real parts, contribute appreciably (Figure 1E, red‐colored small circles).

Such low‐dimensionality is not altered even if during learning the lRNN receives noisy‐contaminated observables or some of them are not provided as input. Indeed, the autonomous lRNN continues to predict accurately the system's future also under these conditions, although the less relevant poles move away from the imaginary axis (missing match between small and large circles).

### 2.2 Stochastic lRNNs Successfully Forecast fMRI Time Series

**FIGURE 2:**
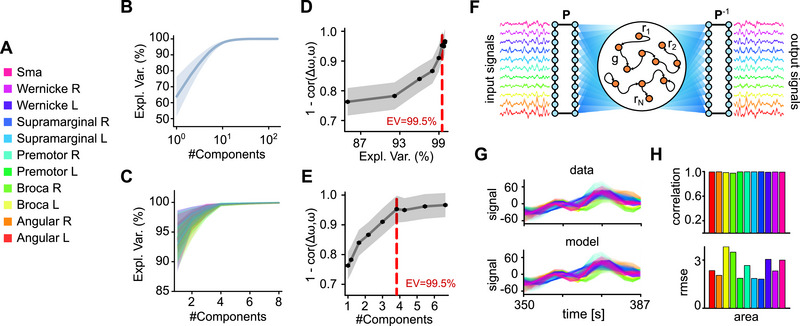
Compression and prediction of resting state fMRI activity. (A) Color legend associated with the 11 cortical areas taken from the language network. (B) Explained variance (EV) from PCA of the fMRI BOLD signals of the voxels in the language network. Solid line, average across the 20 considered subjects. Shaded area, standard deviation across subjects. (C) EV of the PCA for each cortical area. Colors as in (A). (D,E) 1 minus the correlation between the residuals from the PCA reconstruction (Δω) and the original data (ω) per single area in the last (unseen) 37.5 s. A value equal to one means that the remaining information is just white noise. Vertical dashed lines, explained variance (D) and number of first PCs (E) required to reach the EV of 99.5%. (F) Schematic design of the used lRNN. The input from each area is compressed to have an EV of 99.5%. P is the matrix operating this linear transform leading to an average of 44 observables. The lRNN output reconstructing these observable is then uncompressed applying $P^{-1}$. (G) Example of forecasted (bottom) and original (top) activities during the test window (last 37.5 s). Each curve is the BOLD signal of a voxel with color indicating the cortical area of belonging. (H) Forecast performances of the post‐learning autonomous lRNN per area. Correlations between predicted and experimental activity (top) and root mean square error (bottom).

To explore the applicability of the RC with lRNN, we examined time series data from a real‐world physical system. We considered the brain activity of 20 healthy subjects at rest (i.e., in a state of quiet wakefulness), measured through fMRI. The experimental time series was derived from the BOLD signals recorded from a subset of voxels, which serve as proxies for neuronal activity across 11 cortical areas in the language network (Figure 2A, see Methods Section).

The BOLD time series were preprocessed using principal component analysis (PCA) to reduce redundancy and achieve dimensionality reduction, which was found to be relatively high (see Figure 2B, C). Specifically, we assessed the correlation between the information lost during dimensionality reduction and the actual signals. Our analysis revealed that the first principal components (PCs), which explain 99.5% of the variance (Figure 2D,E), excluded a residual activity that was indistinguishable from white noise. Based on this criterion, we determined that, on average, only four PCs per cortical area should be considered. Consequently, the average dimensionality of the dataset was M≈44 PCs per subject (refer to Methods Section for details). This relatively high‐dimensional time series served as the input for the lRNN. The readout synaptic matrix $W^{\mathrm{out}}$ was computed over the first 280 s (Figure 2F).

The decision to utilize a transformation of brain activity that results in information loss may appear counterproductive. However, projecting onto the first PCs was expected to be less sensitivity to the uncertainties inherent in the measured BOLD signals. By employing these first PCs as inputs, we provided the lRNN with denoized representations of brain activity. Moreover, thanks to the echo state property [4, 31, 32], the missing information regarding the state of the observed system can be effectively recovered through the dimensional embedding that the lRNN performs.

As previously described, with the learned $W^{\mathrm{out}}$ – which varies from subject to subject – we established a closed loop between the input and output of the lRNN, effectively creating an autonomous digital twin of the brain network under investigation. This approach yielded a remarkable overlap between the reconstructed and measured time series (see Figure 2G for an example subject). At the population level, we evaluated the forecasting performance by calculating the correlations between replicated and experimental BOLD signals, as well as the root mean square errors (RMSEs) for the final unseen 37.5 s of the time series (Figure 2H). From this perspective, the inferred digital twin demonstrated high‐quality reconstruction of brain activity, further validating its effectiveness.

The lRNN dynamics described thus far is dissipative, meaning that the state of the network ultimately relaxes to 0. However, brain activity associated to the BOLD signals fluctuates without rest. To reconcile this discrepancy in our digital twin, we incorporated an endogenous noise into each network unit. This modification introduces a generative mechanism that sustains activity over time. More specifically, the autonomous dynamics of the inferred (i.e., learned) lRNN dynamics is a multi‐variate Ornstein‐Uhlenbeck process

(9) $$\tau dr=(\tilde{W}-I)rdt+d\eta \;$$

where η(t) is the the vector of Gaussian white noise each unit of the network independently receives ($\left\langle d\eta_{j}\left(t\right)d\eta_{k}\left(t^{\prime }\right)\right\rangle =\gamma^{2}\delta_{jk}\delta \left(t-t^{\prime }\right)$) with noise intensity γ. Note that, since the system is linear, this formulation does not change the theoretical framework derived above; in this case, it applies to the expectation values E[r] and $E\left[\omega \right]=W^{\mathrm{out}}E\left[r\right]$.

### 2.3 Stochastic lRNN Outperforms Hankel DMD on Filtered Datasets

**FIGURE 3:**
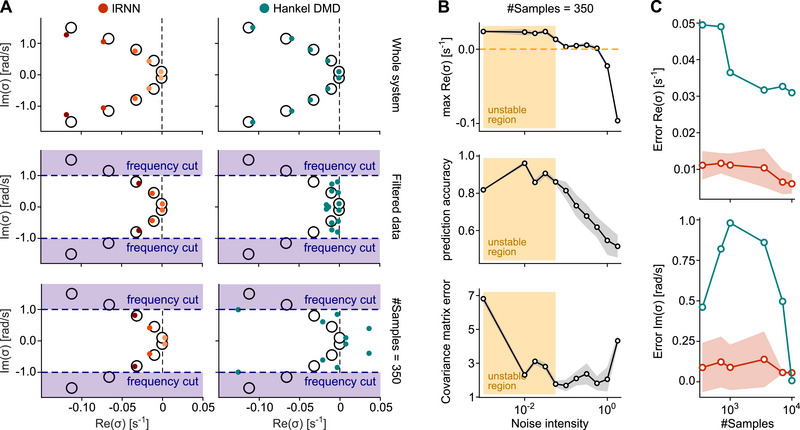
Optimal stochastic lRNNs recover filtered dynamics more accurately than Hankel DMD. (A) Comparison of estimated spectra for a 10‐D multivariate Ornstein‐Uhlenbeck process: lRNN with N=2000 units and endogenous noise (red dots) versus Hankel DMD (green dots), with the ground‐truth spectrum (black circles). From top to bottom: (i) training on the full unfiltered dataset; (ii) training on full low‐pass filtered dataset with cut‐off frequency at $1\;\mathrm{rad}s^{-1}$; (iii) training on a low‐pass filtered dataset of 350 samples. (B) Tuning of lRNN noise intensity based on three metrics (mean over 20 noise realizations): the maximum real part of the inferred poles $\sigma_{k}$; correlation between predicted and true signals; and mismatch between the long‐run reproduced covariance and the training‐set covariance. Yellow region: unstable regime where maxReσk is significantly greater than 0 (threshold: >3 standard deviations). (C) Estimation error of the real and imaginary parts of the first three eigenvalues (sorted by real part) for lRNN (red) and Hankel DMD (green) as a function of available number of samples in the filtered dataset. Shadings: standard deviation across the 20 noise realizations.

A key advantage of this stochastic formulation emerges when fitting lRNNs to filtered data, a standard step in fMRI preprocessing pipelines [39, 40]. Figure 3 compares spectral recovery in stochastic lRNNs to Hankel dynamic mode decomposition (HDMD), a widely used autoregressive alternative [41, 42] (see Methods Section), in the task of learning a 10‐D multivariate Ornstein‐Uhlenbeck process. HDMD recovers the full distribution of poles when trained on unfiltered long datasets (green dots in Figure 3A, top). However, applying a low‐pass filter induces an artificial collapse of the poles toward the imaginary axis (Figure 3A, middle and bottom), thereby obscuring the true dissipative structure of the underlying dynamics. This structure is not recovered even when performing singular‐value decomposition with a truncated rank that excludes noisier dimensions (Figure S1). By contrast, lRNNs with appropriately tuned noise (red dots in Figure 3A) preserve accurate spectral recovery even under filtering and in the small‐dataset setting, closely matching the ground‐truth eigenvalue distribution.

This robustness depends on selecting an optimal noise intensity. Figure 3B shows noise‐level tuning using the maximum real part of the poles (top), prediction accuracy (middle), and long‐run covariance matching (bottom). Increasing noise level strengthens the effective regularization during learning, which progressively reduces the real parts of the inferred poles by pushing them back toward the original random bulk. Focusing on the stable regime (white region in Figure 3B), where the maximum real part is compatible with having negative values (threshold: >3 standard deviations), the optimal noise level maximizes short‐horizon prediction accuracy while best matching the signal's statistical properties. In particular, (i) we computed the correlation between predictions and data over the final 20% of the dataset, and (ii) we ran the autonomous and stochastic lRNN for an additional duration equal to the training window (the first 80% of the dataset) and measured the discrepancy between the resulting covariance matrix and that of the training data.

An intermediate noise regime, near the stability boundary, tends to optimize all three metrics. Excessive noise intensities degrade prediction and prune too many modes, whereas insufficient noise fails to infer stable, realistic BOLD fluctuations eventually leading to divergence. This sweet spot enables consistent estimation across sample sizes, as reflected in the pole estimation errors in Figure 3C (red curves), whereas HDMD (green curves) continues to produce erroneous estimates.

Such results encapsulate the central distinction between the lRNN and standard AR/DMD approaches in their modeling assumptions. These methods estimate a linear map for the conditional mean evolution. Our lRNN instead fits a dissipative, noise‐driven, higher‐dimensional process. For heavily low‐pass filtered signals such as BOLD, “best linear predictor” methods may converge to spectra that describe the smoothed average evolution (effectively a Fourier‐like decomposition of the filtered signal), whereas the stochastic lRNN is constrained to produce a stable stochastic surrogate, which is precisely why spectral recovery remains robust under filtering and in the small‐dataset regime.

### 2.4 Stochastic lRNN as a Digital Twin of Brain Activity

**FIGURE 4:**
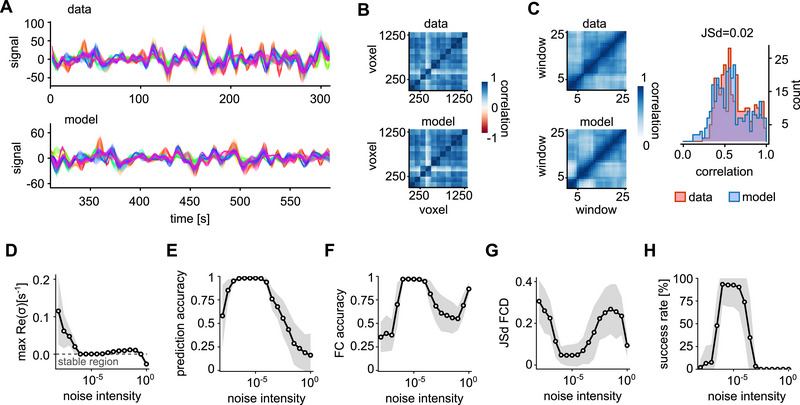
Endogenous noise in lRNN is needed to replicate brain activity. (A) Comparison of actual brain activity (top) with the corresponding output from a trained autonomous lRNN over the subsequent prediction interval (bottom). (B,C) Analysis of functional connectivity (FC) and functional connectivity dynamics (FCD) computed from the activity depicted in (A). Right, distribution of FCD values for lRNN‐generated and exeprimental time series with the related Jensen‐Shannon distance. (D–G) Features and performance statistics of inferred lRNNs across subjects varying the amplitude of endogenous noise: maximum pole real part (D), correlation between data and model prediction (E), FC correlation with actual data (F), and Jensen‐Shannon distance between FCDs (G). (H) Success rate in reproducing above‐threshold performances of the features in panels (E–G) (see main text for details).

To validate the effectiveness of this method in accurately replicating brain activity (low‐pass filtered and with a limited number of samples), we numerically integrated the network dynamics for an additional 387.5 s (Figure 4A). We then compared the functional connectivity (FC) – defined as the correlation between BOLD signals of all possible voxel pairs – calculated from this simulation to that obtained from the experimental data (Figure 4B). Furthermore, the temporal evolution of the FC, referred to as functional connectivity dynamics (FCD), closely aligned with the experimental observations. This similarity was quantified by the Jensen‐Shannon distance (JSd) between the distribution values of the associated matrices (Figure 4C).

Finally, we conducted a comprehensive analysis of the optimal intensity of noise across all subjects (Figure 4), focusing on the accuracy of BOLD signal forecasting and the lRNN capability to reproduce both FC and FCD. To evaluate the quality of these aspects simultaneously, we introduced a success rate defined by applying fixed thresholds to the performance metrics: accuracy >80%, FC correlation >85% and JSd<0.1. Additionally, we assessed the stability of the inferred lRNN by examining the maximum real part of the poles $\sigma_{k}$ (Figure 4D). According to what is shown in the previous Subsection, increasing the noise intensity during inference progressively shifts the maximum real part of the poles leftward, eventually making it negative. This spectral drift enforces dissipativity, thus preventing marginally stable or weakly damped solutions, and stabilizes trajectories, thereby improving out‐of‐sample predictions. This results further demonstrate that noise in our lRNN acts as a constructive element, essential for recovering the correct stochastic dissipative dynamics that best match the experimental time series. This analysis enabled us to identify a common optimal intensity γ of endogenous noise across subjects with a standard deviation of $10^{-6}$ (Figure 4H). Interestingly, the existence of an optimal noise displays some similarities with the resonance phenomenon found in other machine‐learning studies [43], which in our case robustly emerged in all the subjects.

### 2.5 Dynamical Properties of the Digital Twin

**FIGURE 5:**
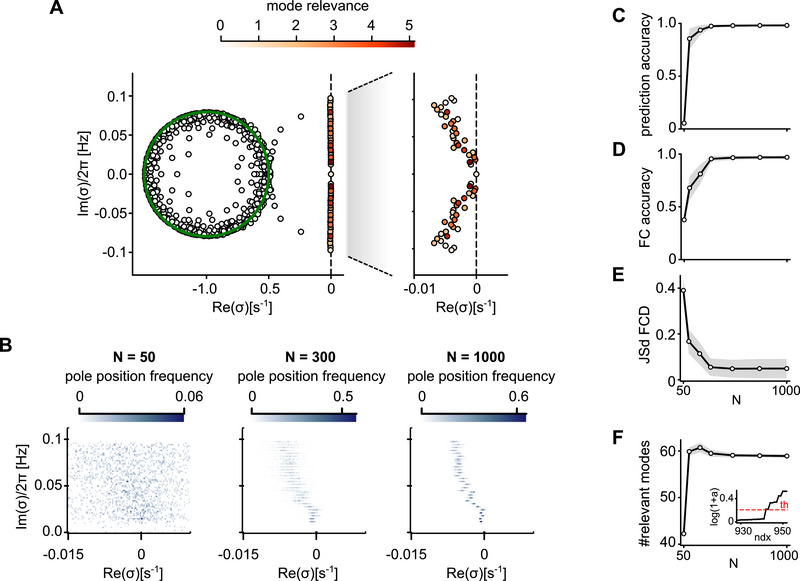
Convergence of the mode decomposition with increasing lRNN size. (A) Mode decomposition of a single cortical area for an example subject. Eigenvalues of $\tilde{W}$ are color‐coded as in Figure 1E. (B) Distribution of eigenvalues for three distinct network sizes N, analyzed across 100 randomly sampled lRNNs. (C) Accuracy of forecasted activity. D, E) Reproduction fidelity of FC (D) and of FCD (E). (F) Number of relevant modes (i.e., with relevance greater than 5%). Inset, sorted mode relevance and related threshold (dashed line). Gray shadings and black lines, standard deviation and mean across subjects, respectively.

We showed that RC with lRNNs can effectively reproduce resting state fMRI activity. Each simulated voxel can be described as a decomposition of linearly evolving modes. The dynamical properties of this decomposition can be effectively represented by the poles $\sigma_{k}$ of the Laplace transform of the inferred lRNN, which are the eigenvalues ${\tilde{\lambda }}_{k}$ of the learned synaptic matrix $\tilde{W}$. This spectrum of eigenvalues is shown in Figure 5A for an example subject. For each of the modes, the relevance is averaged across all voxels and it is color coded, showing a pattern similar to what seen in Figure 1E where only a subset of poles are moved towards the imaginary axis. These poles are the most relevant and display a specific organization like a rotated parabola. The modes with the highest relevance (i.e., the darkest) appear to be distributed at specific frequencies falling into the range of infra‐slow oscillations (<0.1 Hz), a typical footprint of the resting state and of the unconscious brain activity [44, 45, 46, 47]. As a representative example, in Figure 5A, the most relevant mode is one of the most persistent, characterized by a relatively small $\text{Re}\;\sigma_{k}$, and exhibits a resonant frequency of approximately 0.02 Hz.

Subsequently, we investigated the stability of this representation exploring how the spectrum of $\tilde{W}$ and the mode relevance change according to the number N of units in the lRNN. As shown in Figure 5B, the density of eigenvalues in the complex plane (Reσ,Imσ) becomes less and less scattered with increasing network size. For clarity, and to save space, here we plotted only one half of the complex plane; the other half follows by complex‐conjugate symmetry, which holds by having real‐valued synaptic matrix. Besides, all the performance measures converged to fixed values corresponding to a high quality of the data replicate by the digital twin (Figure 5C‐E), proving that a finite size lRNN can reach asymptotically high performances. Intriguingly, as convergence is approached the average number of relevant modes stands at about 60 independent components (Figure 5F). This number is significantly higher than the number of experimental PCs provided as input to the lRNN (44 on average). Thus, brain activity replicated by the inferred lRNN appear to live in a latent state space whose dimensionality is larger than the one determined by its observation.

**FIGURE 6:**
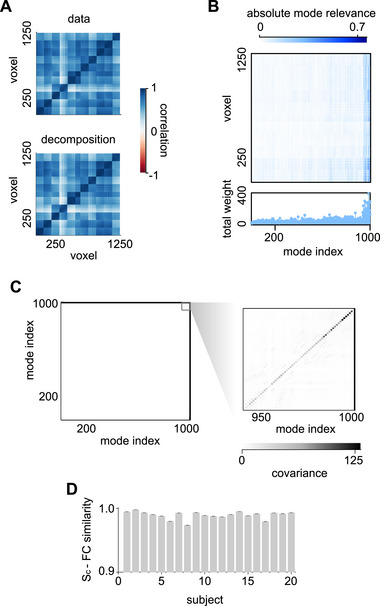
Low‐dimensional mode decomposition explains functional connectivity. (A) Functional connectivity of an example subject (top) and cosine similarity (bottom) between rows of the matrix Z mapping eigenmode projections to voxel‐wise BOLD activity from the inferred lRNN with N=1000 units (see main text). (B) Absolute mode relevance $|Z_{jk}|$ for the same lRNN together with the total weight $\sum_{j}\left|Z_{jk}\right|$ of each of the N modes (bottom). Modes are sorted according to the real part of the associated eigenvalue ($\text{Re}\;{\tilde{\lambda }}_{k}$). (C) Covariance matrix of the eigenmode projections $v_{k}\left(t\right)$ (see Equation (7)) during the extended autonomous phase (387.5 s post learning in Figure 4A‐bottom). Right, zoomed‐in view on the slow and most relevant modes. (D) Correlation between the upper diagonal elements of the FC and the cosine similarity ($S_{C}$) of the rows of Z for each subject. Averaged over 100 noise realizations. Error bars, SEM.

Functional connectivity illustrated in Figure 6A‐top for an example subject is then fully replicated by a limited number of independent modes of the inferred (and stochastic) lRNN. This effective copy occurs even though the dimensionality of the latent state space of the digital twin is significantly smaller than the number of voxels encompassing the examined language network. To gain a deeper understanding of this result, we ‘opened the box’ by examining the linear transformation $Z\equiv P^{-1}\Xi \in R^{L\times N}$. According to Equation (7), this matrix facilitates the mapping of the N eigenmode projections v(t) onto the L‐dimensional voxel‐wise BOLD activity, represented as x(t)=Zv(t). The absolute values of the elements in this matrix are displayed in Figure 6B for the same subject. Unsurprisingly, the most significant contributions arise from the slowest (rightmost) modes, specifically those with the largest, albeit still negative, $\text{Re}\;\sigma_{k}$. Additionally, by measuring the covariance between the eigenmode projections $v_{k}\left(t\right)$, it becomes evident from Figure 6C that they are largely uncorrelated. Only about 60 of the slowest modes exhibit significant variability, as indicated by the dark diagonal elements. This empirical evidence suggests that functional connectivity can be estimated directly using the cosine similarity $S_{C}$ between the rows of the matrix Z (see Methods Section):

(10) $${\mathrm{FC}}_{jk}\approx S_{C}\left(\left\langle Z_{j}\right|,\left\langle Z_{k}\right|\right)\;$$

In Figure 6A‐bottom, the similarity matrix is presented, showcasing a remarkable overlap with the experimental functional connectivity FC. It is important to note that in computing Z, we considered only the columns of Ξ associated with the relevant modes. This further confirms that the remaining N−60 degrees of freedom are nearly irrelevant.

### 2.6 lRNN as a Proxy to Characterize Subjects and Brain Areas

**FIGURE 7:**
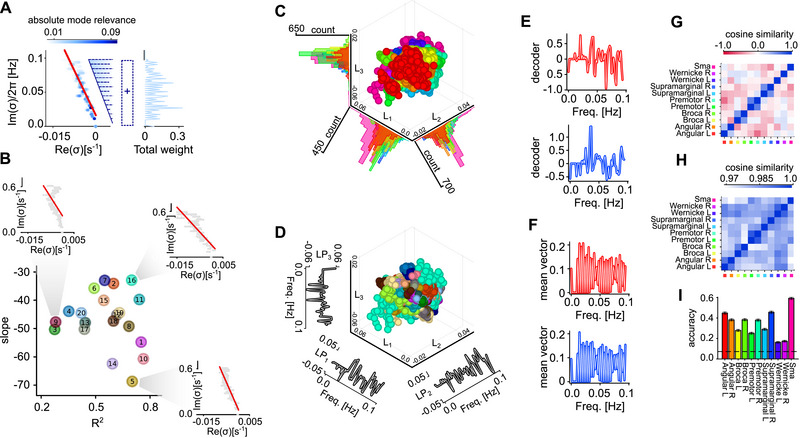
Characterization of subjects and brain areas. (A) Left, Spectrum of relevant eigenmodes for a sample subject. A linear regression (red line) is performed between the real and imaginary parts of the poles, $\sigma_{k}$, with the slope serving as the first feature that characterizes the subject. The coefficient of determination, $R^{2}$, from this regression represents the second feature. Right, Frequency vector, i.e., the histogram of $\text{Im}\;\sigma_{k}$ each counted with multiplicity given by the averaged absolute mode relevance across voxels in a single brain area: $\left\langle \right|\Xi_{jk}{\left|\right\rangle }_{j\in \mathrm{BA}}$. (B) Slope of linear regression and $R^{2}$ of the 20 subjects in the dataset. Insets: eigenmode spectra of three representative subjects with the related linear regressions (red lines, see main text for details). (C) First three latent variables derived from the linear discriminant analysis (LDA) applied to the frequency vectors of 11 brain areas across all 20 subjects. The densities of circles for each latent variable are also plotted. For each subject, 100 lRNNs are inferred by sampling different endogenous noise in their stochastic dynamics, resulting in a total of 22,000 circles. Colors code brain areas according to Figure 2A. (D) Same as (C) but with circles colored according with the subject color as in (B). Composition of the three latent variables ${\mathrm{LP}}_{1}$, ${\mathrm{LP}}_{2}$, ${\mathrm{LP}}_{3}$ are show on the side of their respective axes. (E,F) Linear decoders (E) discriminating the different areas in the frequency‐vector space and average frequency vectors (F) for two sample brain areas (red and blue). (G,H) Cosine similarity between linear decoders (G) and between frequency vectors (H) per area averaged across subjects. (I) Accuracy of decoders along the training set. Dashed black line highlights the random chance level separation.

Given that inferred lRNNs reliably replicate observed brain activity and their modal decomposition defines their dynamical properties, a critical question emerges: Can lRNNs serve as proxies for understanding the similarities and differences among subjects and cortical areas? To address this, we characterized the spectrum of relevant eigenvalues by focusing on two key features: the linear relationship between their real and imaginary parts, and the overall significance of each oscillation frequency, as illustrated in Figure 7A. The slope of this linear relationship indicates the correlation between decay times and oscillation frequencies, while the coefficient of determination ($R^{2}$) assesses the goodness of fit for this linear model. In this 2D parameter space, subjects are systematically distributed among three distinct regions (Figure 7B). Those with high $R^{2}$ values display either steep or shallow slopes, whereas subjects with low $R^{2}$ values indicate a poor linear fit and suggest the presence of persistent isolated high‐frequency oscillatory modes. Within this representational space, spectra characterized by steeper slopes (located at the bottom) are associated with poles that are closer to the imaginary axis. This suggests that those lRNNs (and consequently subjects) exhibit longer relaxation time scales, which may be linked to brain activity approaching a critical point where the resting state could become unstable and display more complex dynamics.

Shifting our focus to the frequency vectors derived from the inferred lRNN, we sought statistical regularities that reveal invariant features associated with individual brain areas and subjects. The frequency vector for a given brain area in a subject is represented by the histogram of $\text{Im}\;\sigma_{k}$ from the corresponding lRNN, each counted with multiplicity proportional to its absolute mode relevance averaged across the voxels in that area: $\left\langle \right|\Xi_{jk}{\left|\right\rangle }_{j\in \mathrm{BA}}$. We then performed a linear discriminant analysis (LDA) in the frequency‐vector space to identify patterns that effectively discriminate between different brain areas (Figure 7C). It is important to emphasize that the frequency vectors used in this analysis were obtained from 100 independently inferred lRNNs for each subject. This approach ensured a fair and robust discrimination analysis, as it explicitly incorporates the unavoidable variability introduced by the stochastic lRNN inference process, although (as shown in Figure 4B) the distribution of poles is already highly stable for the chosen network size. Despite originating from distinct subjects, the resulting data points clustered according to their respective brain areas. It is important to note that the compositions of the latent variables ($L_{1}$, $L_{2}$, and $L_{3}$) correspond to specific frequency patterns (Figure 7D). Among these, the pattern associated with $L_{1}$ appears most relevant, as data points on this linear manifold are distinctly clustered based on the brain area of belonging (Figure 7C).

To further isolate the frequencies at which each brain area exhibits unique dynamics, we computed the corresponding linear decoders (see Methods Section). The elements of the coefficient vectors characterizing each decoder are assigned high absolute values if the associated frequency plays a crucial role in distinguishing that area from others (Figure 7E). The resulting patterns are markedly distinct from the average frequency vector, which, in contrast, reveals a continuum of relevant frequencies (Figure 7F). In fact, the frequency vectors show significant similarities, making it challenging to differentiate between areas even if they are located in different hemispheres (Figure 7H). In contrast, the decoders exhibit a lower degree of similarity, except when comparing the corresponding areas of both hemispheres (Figure 7G). Interestingly, there is an exception to this trend: the decoder for the right Wernicke area appears to highlight frequencies that differ from those identified for the same area in the left hemisphere.

Further insights into brain lateralization are revealed by examining the accuracy of the decoders. Specifically, it is more difficult to linearly recognize left hemisphere patterns by this method for the Broca, premotor, and supramarginal areas (Figure 7I). This asymmetry likely reflects the well‐established left‐lateralization of the resting‐state language network in right‐handed individuals. In particular, the inferior frontal gyrus (including Broca's area), the supramarginal gyrus, and the premotor/pre‐supplementary motor areas exhibit stronger intrinsic left‐hemisphere connectivity at rest [48, 49, 50]. As a consequence, their BOLD time courses tend to covary more strongly within the left hemisphere, making these regions harder to dissociate and likely explaining the reduced decoder accuracy compared to their more independent right‐hemisphere homologues. It is also worth noting that Wernicke's area appears as the most difficult region to recognize, thereby explaining the decoder dissimilarity observed in Figure 7G. Importantly, despite these heterogeneous coupling strengths across subnetworks, stochastic lRNNs can still be robustly inferred, demonstrating their ability to capture the effective dynamics of resting‐state activity even in the presence of strong intra‐network coherence.

In short, these findings demonstrate that inferred lRNNs can serve as powerful proxies to characterize the unique dynamics of subjects and areas of the brain, capturing their specific dynamical footprints.

## 3 Discussion

The successful application of linear recurrent neural networks within the reservoir computing framework represents a significant methodological contribution to model resting‐state fMRI data. Our results show that lRNNs effectively capture brain dynamics, suggesting that resting‐state activity can be approximated as a linear system in a high‐dimensional space, supporting recent studies advocating for linear models in this context [51]. Our approach provides valuable and easily accessible insights into the functional connectivity structure as similarity patterns between vectors associated with the eigenmode decomposition of the inferred lRNN. This low‐dimensional description of the whole brain activity moves the focus to the mode's subspace offering a normative framework where brain areas and inter‐subject comparisons are more straightforward.

Notably, the mode dynamics described in Equation (6) provides an equivalent linear representation for the observables of the system under investigation – namely, the single‐subject voxel‐wise BOLD activity. The resulting vector of all time derivatives (see Methods Section) serves as an alternative descriptor of the system's state [52], potentially encapsulating the trajectory evolution at any given time. This concept is reminiscent of the dynamic mode decomposition with Hankel matrix [41, 42], but with a significantly reduced dimensionality constraint. However, we showed that Hankel‐DMD in practice tends to converge toward a Fourier‐like representation when the data undergo standard frequency‐band filtering (as commonly done in fMRI analysis pipelines), while our lRNN formulation can still recover the correct continuous‐time spectrum, including nonzero decay rates. In filtered conditions, Hankel‐DMD often yields poles whose real parts collapse toward 0, effectively favoring purely oscillatory modes and obscuring the dissipative structure of the underlying dynamics (Figure 3). This result also stems from considering an optimal amount of memory‐less fluctuations integrated into the lRNN units’ endogenous activity. This feature enables effective modeling of non‐informative fast fluctuations in BOLD activity without requiring inference of additional high‐frequency, fast‐decaying modes, thereby reducing the dimensionality of the latent state space visited by the deterministic components of the inferred lRNNs. This characteristic makes the presented method particularly advantageous and flexible, allowing the inference of lRNN‐based digital twins at the single‐subject level using a limited amount of experimental data. In contrast, several recent approaches have applied deep reinforcement learning (DRL) to fMRI timeseries or brain‐network analysis [53, 54]. These DRL‐based methods typically require reward‐driven optimization strategies and substantially larger training pipelines [55]. Differently from these approaches, we show that our stochastic lRNN framework achieves accurate, robust, and interpretable data‐driven modeling by ‘learning’ only a limited number of readout weights through straightforward linear regression, similarly to classical echo state networks [4].

The reported results have the potential to open up several avenues for future research. Indeed, the possibility offered to represent the single‐subject as a point into a low‐dimensional map – i.e., a landscape (Figure 7B) – allows in principle to follow in time its drift in longitudinal studies like those focused on neurodegenerative diseases [56] or aging [57, 58, 59]. Here, the novelty is the fully data‐driven nature of the approach to build such a landscape of subjects compared to those relying on model‐driven inferences [60]. Characterizing the trajectory followed in this landscape by each subject could provide valuable insights into the alterations in functional connectivity and dynamics. For instance, the emergence of unstable modes may be linked to transitions into pathological states of a specific neurological disease. By perturbing the system with specific and personalized rehabilitative or pharmacological treatments that aim to suppress these “pathology‐related” modes, we may gain a means to intervene and reduce the occurrence of such transitions, ultimately helping to tailor optimal therapeutic strategies [28, 61].

In line with this, as in classic digital twins, the inferred lRNN can incorporate any additional available information about the system simply as new input. An external stimulation can potentially act as a selector for different dynamical regimes, allowing for two distinct representations with a single network [62]. The functional connectivity itself could be a proxy for state transition. Indeed, a significant change in functional connectivity might indicate that the decomposition has also changed, suggesting that the observed system is close to a different equilibrium point [63]. These changes can be tracked following the distribution of poles that characterize the autonomous dynamics of the inferred linear RNN. This is the case for transitions in global brain states – such as those governing the sleep‐wake cycle – that arise from network destabilization, a hallmark of criticality [64, 65, 66]. Under these conditions, pole distributions correlate with longer relaxation time scales and steeper slopes (as illustrated in Figure 7B), possibly underlying previously observed state‐dependent spectral signatures [67]. This metastable dynamics results in broader excursions within the latent state space of neural activity, making predictions of future BOLD activity more challenging for the inferred lRNN. The quality of lRNN predictions can then serve as a proxy for dynamical stability, akin to findings in intracortical local field potentials (LFPs) of macaque monkeys during anesthesia‐induced transitions between wakefulness and unconsciousness [68].

While this study demonstrates the efficacy of lRNNs in modeling resting‐state brain activity, several limitations must be acknowledged. The assumption of linearity, although supported by our results, may not fully capture the complexity of brain dynamics under all conditions. Nonlinear phenomena, transient states, and the influence of external stimuli could require more sophisticated modeling approaches [14, 69]. Alternatively, the apparent suitability of resting‐state activity being well represented by a stationary multivariate Ornstein‐Uhlenbeck process (i.e., Brownian motion) [70, 71] may reflect an intrinsic limitation of the BOLD signal in conveying detailed neural dynamics. Indeed, this hemodynamic‐related signal acts as a lumped observable that inevitably linearizes the spiking activity of relatively large neuronal assemblies [51, 72]. The coexistence of endogenous noise with the relatively high‐dimensionality of the BOLD time series can give rise to a multivariate Brownian motion exhibiting a rich repertoire of restless spatiotemporal modes [71]. These are captured in our lRNN by relatively wide distributions of poles. Within this linear stochastic system, noise spontaneously excites its normal modes, eliciting co‐fluctuating patterns closely linked to the functional connectivity measured in rs‐fMRI. Interestingly, this structured stochasticity may, at least in principle, exhibit similarities to turbulence [73, 74].

However, assuming that all voxel activities fluctuate around a single fixed point imposes a strong constraint on the brain's computational repertoire. Neural computation is thought to emerge from trajectories evolving across landscapes rich in saddles and metastable states [75, 76], whereas lRNNs cannot, by construction, represent the latent state space of a genuinely multistable system. Nonetheless, multistability could in principle be handled by partitioning the state space into quasi‐linear regions, each represented by a local lRNN, as in piecewise‐linear approaches [77], or by allowing unstable modes when external inputs drive transitions, similar to recent modeling of cortical trajectories [78].

Besides this modeling limitation, our results raise an important question: how can we reconcile the evidence that resting‐state fMRI activity is well described by stochastic lRNNs with the necessity of nonlinear dynamics for brain computation? A tentative answer is that, during rest, endogenous fluctuations have a magnitude comparable to the deterministic excursions of neural activity associated with relaxation dynamics. Consequently, the nonlinear components of brain activity may only become apparent when the system is pushed far from equilibrium – such as when the brain engages in cognitive functions like motor or perceptual decision‐making. A promising direction for future work would then be to test this hypothesis by assessing whether an effective lRNN can be inferred from BOLD time series recorded while subjects engage in such cognitive tasks.

All these considerations are particularly relevant because the metastable neural dynamics are tightly linked to both healthy and pathological brain function. In this framework, a failure to infer a stochastic lRNN that faithfully reproduces resting‐state BOLD signals can be interpreted as a quantitative marker of changes in metastability. Such failures in inferring an effective lRNN may therefore serve as a sensitive biomarker of brain dysfunction, ultimately aiding the identification of disease‐related alterations in dynamical coordination (e.g., schizophrenia, depression, Alzheimer's disease, epilepsy; see [79]).

## 4 Methods Section

### 4.1 Dataset Description

#### 4.1.1 Subjects

The study included 20 healthy, right‐handed subjects (mean age ± SD = 37±12; 7 females, 13 males) with no history of neurological disorders. The study was approved by the Institutional Review Board at Memorial Sloan Kettering Cancer Center, and informed consent was obtained from each participant. During the resting‐state condition, subjects were instructed to lie in the scanner, keep their eyes open, try to think of nothing in particular, and maintain fixation on a central cross on the screen.

#### 4.1.2 MRI Methods

A GE 3T scanner (General Electric, Milwaukee, Wisconsin, USA) and a standard quadrature head coil was employed to acquire the MR images. Functional images covering the whole brain were acquired using a (T2*)‐weighted imaging sequence sensitive to blood oxygen level‐dependent (BOLD) signal (repetition time, TR/TE = 2500/40 ms; slice thickness = 4.5 mm; matrix = 128 × 128; FOV = 240 mm; volumes = 160). Functional matching axial T1‐weighted images (TR/TE = 600/8 ms; slice thickness = 1 mm) were acquired for anatomical co‐registration purposes.

#### 4.1.3 Data Preprocessing

Functional MRI data were processed and analyzed using the software program Analysis of Functional NeuroImages (AFNI; Cox, 1996). Head motion correction was performed using 3D rigid‐body registration. The first volume was selected to register all other volumes. The first volume was chosen because it was acquired before the anatomical scan. Both task and resting state fMRI scans were monitored using a real time post‐processing software BrainWave (BrainWave RT, Medical Numerics, Germantown, MD) to monitor brain activity and the head motion. For subjects showing severe head motion over time, generally, the scan was repeated. For small head motion (less than 2 voxel size), a motion correction algorism (iterated linearized weighted least squares) considering three translation and three rotation parameters against a reference volume was applied. The obtained six parameter time courses were also integrated in the statistical analysis to regress out residual motion‐correlated artifactual voxels. Spatial smoothing was applied to improve the signal‐to‐noise ratio using a Gaussian filter with a 6 mm full width of half maximum. Corrections for linear trend and high‐frequency noise were also applied. Resting‐state data requested some more preprocessing steps. They were corrected for head motion by regressing head motion data and the first five principal components of the white matter and CSF signals. They were also detrended, demeaned, and band‐pass filtered (frequency range 0.01–0.1 Hz). All fMRI data were registered to the standard space (Montreal Neurological Institute MNI152 standard map).

### 4.2 fMRI Dimensionality Analysis

Principal Component Analysis (PCA) is a widely used dimensionality reduction technique used to transform a dataset into a lower‐dimensional space while preserving most of the information in the original data [80]. In the context of the given problem, PCA was applied to the set of BOLD signals to reduce redundancy and keep only the relevant information.

The PCA‐based dimensional reduction of BOLD was carried out based on the initial 280 s (learning period) of each subject's data. Referring to Figure 2, the number of principal components taken into account were those capturing 99.5% of the variance in the time series of each area. The resulting transformation was represented by a block matrix $P_{0}$, where each block's columns $p_{i}$ correspond to the eigenvectors of the covariance matrix of the respective cortical area:

(11) $$P_{0}=\left[\begin{array}{ccc} p_{1} & \cdots & 0\\ \vdots & \ddots & \vdots \\ 0 & \cdots & p_{11} \end{array}\right]$$

The output undergoes orthogonalization through an additional PCA step ($P_{1}$) utilizing all components. Subsequently, normalization was performed by applying the diagonal matrix N, which contains the reciprocals of the maximum values of the compressed signals observed during the learning period. This approach ensured that all currents enter the reservoir with the same maximum strength. The final transformation matrix P is computed as:

(12) $$P=NP_{1}P_{0}$$

While the antitransformation matrix $P^{-1}$ is calculated as:

(13) $$P^{-1}=P_{0}^{T}P_{1}^{T}N^{-1}$$

The data was effectively reduced and then restored, ensuring the preservation of essential information while reducing dimensionality for the analysis. The decomposition for voxel activity was derived by applying $P^{-1}$ to the decomposition matrix for the actual network inputs.

### 4.3 Koopman Operator

The discrete‐time Koopman operator $K^{1}:F\to F$ is a linear operator defined in an infinite‐dimensional space F of observables of the system state [19, 20, 81]. The operation of the Koopman operator on an observable g is described by the equation:

(14) $$K^{1}g\left(x_{t}\right)=g\left(x_{t+1}\right)\;.$$

Here, x represents a point in the state space. The eigenfunctions of the Koopman operator were potential observables of the system themselves, and had the peculiar property of evolving linearly in time:

(15) $$K^{1}\psi_{k}\left(x_{t}\right)=\psi_{k}\left(x_{t+1}\right)=\lambda_{k}^{1}\psi_{k}\left(x_{t}\right)\;$$

These eigenfunctions $\psi_{k}\left(x_{t}\right)$ constitute a basis of the space F, enabling the representation of all the possible observables of the system as a linear combination of linearly evolving functions.

In general there existed a family of Koopman operators, $K^{t}$, that advances a function forward by a time t:

(16) $$K^{t}g\left(x\right)=g\left(S^{t}\left(x\right)\right)\;.$$

Here, $S^{t}$ denotes the time flow operator, with $x\left(t\right)=S^{t}\left[x\left(0\right)\right]$. The infinitesimal generator of the Koopman operator family $Lg=\lim_{t\to 0}\frac{K^{t}g-g}{t}$ is the Lie operator which evaluates the temporal change of observables:

(17) $$Lg\left(x\left(t\right)\right)=\frac{d}{\mathrm{dt}}g\left(x\left(t\right)\right).$$

The family of Koopman operators can then be expressed in this term $K^{t}=e^{Lt}$.

### 4.4 Hankel Dynamic Mode Decomposition (Hankel DMD)

Hankel Dynamic Mode Decomposition was employed to compute a finite‐dimensional approximation of the Koopman spectral properties from the measured observables by applying DMD to a delay‐embedded representation of the time series [41, 42]. Given a sequence of snapshots $\omega \left(t_{k}\right)\in R^{M}$, sampled at constant interval Δt, a block‐Hankel (time‐delay) data matrix of memory length q is constructed as

(18) $$H=\left[\begin{array}{cccc} \omega (t_{1}) & \omega (t_{2}) & \cdots & \omega (t_{K})\\ \omega (t_{2}) & \omega (t_{3}) & \cdots & \omega (t_{K+1})\\ \vdots & \vdots & \ddots & \vdots \\ \omega (t_{q}) & \omega (t_{q+1}) & \cdots & \omega (t_{K+q-1}) \end{array}\right]\in R^{qM\times K}\;$$

whose columns represent delay‐embedded states, while the first block‐row coincides with the original observations. Two embedded snapshot matrices were then defined as $H_{0}=\left[h_{1},\text{…},h_{K-1}\right]$ and $H_{1}=\left[h_{2},\text{…},h_{K}\right]$, where $h_{k}$ denotes the k‐th column of H, and the best‐fit linear map on the embedded space was sought in the form $H_{1}≃KH_{0}$.

As in standard DMD, a truncated singular value decomposition (SVD) of the embedded snapshot matrix was computed as $H_{0}≃U_{r}\Sigma_{r}V_{r}^{T}$, where the truncation rank r was selected according to the retained SVD energy. The reduced operator is then obtained as

(19) $$K=H_{1}\;V_{r}\;\Sigma_{r}^{-1}\;U_{r}^{T}$$

and its eigendecomposition $Kw_{j}=\lambda_{j}w_{j}$ provides the DMD eigenvalues $\lambda_{j}$. The associated generator spectrum is obtained from $\sigma_{j}=\Delta t^{-1}\log\left(\lambda_{j}\right)$.

### 4.5 Reservoir Computing

The reservoir computing approach involved a RNN with fixed and random internal couplings (namely the ‘reservoir’) whose state was fed forward to a second set of ‘readout’ units [4, 31]. In this framework the required ‘echo‐state property’ spontaneously emerges from the RNN collective dynamics. The units composing the RNN are intended to model homogeneous and local neuronal assemblies of a cortical network [82, 83, 84]. Each of the N units in the network has activity state $r_{j}\left(t\right)$ evolving in time as

(20) $$\tau \dot{r_{j}}=\Phi \left(h_{j}\right)-r_{j}$$

with j∈[1,N]⊂Z. The decay time constant τ and the sigmoidal activation function $\Phi (h_{j})$ is the same for all units. Here, we consider the limiting case of weak recurrent coupling, where the activation function can be approximated to a linear function: $\Phi \left(h_{j}\right)=h_{j}$. The synaptic input $h_{j}$ is the weighted sum

$$h_{j}\left(t\right)=\sum_{k=1}^{N}W_{jk}r_{k}\left(t\right)+h_{j}^{\mathrm{in}}\left(t\right)$$

where $W_{jk}$ are the elements of the synaptic matrix (i.e., the internal couplings) $W\in R^{N\times N}$, and $h_{j}^{\mathrm{in}}\left(t\right)$ is the external input received by the unit j. In reservoir computing, the external input is driven by the measured observable ω of the inspected system, which is fed into the network using random synaptic couplings: $h_{j}^{\mathrm{in}}\left(t\right)=W_{j}^{\mathrm{in}}\;\omega \left(t\right)$. Due to the high dimensionality of the trajectories in the RNN state space, a simple linear transformation represented by $W^{\mathrm{out}}$ is often sufficient to accurately map the RNN state into a desired output time series. More precisely, given $R\in R^{N\times T}$ whose k‐th row represents the time series of the k‐th unit during a learning period lasting T time steps, and $\Omega \in R^{M\times T}$ has rows given by the M observables $\omega_{k}\left(t\right)$ to be replicated, the linear map is computed by a ridge regression:

(21) $$W^{\mathrm{out}}=\Omega R^{T}{\left(RR^{T}+\beta I\right)}^{-1}$$

where β is a regularization parameter and $I\in R^{N\times N}$ the identity matrix. The target output coincides with the external input. Then, the resulting linear map can be used to simulate the external stimulation and predict the future steps of the data. This is equivalent to update the synaptic matrix of the reservoir. In particular $W^{\mathrm{in}}\omega \left(t\right)\approx W^{\mathrm{in}}W^{\mathrm{out}}r\left(t\right)$ leading to the autonomous system:

(22) $$\tau \dot{r}=\Phi \left[\tilde{W}r\right]-r\;$$

where $\tilde{W}=W+W^{\mathrm{in}}W^{\mathrm{out}}$ is the updated synaptic matrix.

### 4.6 Linear Recurrent Neural Networks (lRNN) and Autoregressive Models

In the established framework of reservoir computing with linear RNNs (lRNN, i.e., with a linear activation function) by setting as initial condition $r_{k}\left(-\infty \right)=0$, the system Equation (20) has the following solution:

(23) $$r\left(t\right)=\sum_{k=1}^{M}\int_{-\infty }^{t}\omega_{k}\left(t^{\prime }\right)e^{\left(W-I\right)\left(t-t^{\prime }\right)/\tau }|W_{k}^{\mathrm{in}}\rangle dt^{\prime }$$

As above $\omega_{k}\left(t\right)$ denotes the k‐observable measured from the inspected system. Assuming a time $t^{*}$ exists after which a matrix $W^{\mathrm{out}}$ maps the network state to the input data $\omega \left(t\right)=W^{\mathrm{out}}r\left(t\right)$, we can rewrite the above equation as:

(24) $$\omega \left(t\right)=\sum_{k=1}^{M}\int_{-\infty }^{t}\omega_{k}\left(t^{\prime }\right)W^{\mathrm{out}}e^{\left(W-I\right)\left(t-t^{\prime }\right)/\tau }|W_{k}^{\mathrm{in}}\rangle dt^{\prime }$$

By substituting $s=t-t^{\prime }$, we obtain an autoregressive description of the signals:

(25) $$\omega_{j}\left(t\right)=\sum_{k=1}^{M}\int_{0}^{\infty }G_{\mathrm{jk}}\left(s\right)\omega_{k}\left(t-s\right)\mathrm{ds}.$$

Here, $G_{jk}\left(s\right)=W_{j}^{\mathrm{out}}e^{(W-I)s/\tau }W_{k}^{\mathrm{in}}$. These kernels can be expressed in terms of the synaptic matrix spectrum. The diagonal representation of the exponential matrix is given by:

(26) $$e^{(W-I)s/\tau }=\sum_{n=1}^{N}e^{(\lambda_{n}-1)/\tau }|Q_{n}\rangle \langle Q_{n}^{-1}|\;.$$

In this representation, $\lambda_{n}$ denotes the eigenvalues and $|Q_{n}\rangle$ $\left\langle Q_{n}^{-1}\right|$ the outer product between the right and left eigenvectors of W. This representation allows us to rewrite the kernel as the weighted sum over exponentially decaying components:

(27) $$G_{jk}\left(s\right)=\sum_{n=1}^{N}J_{jk}^{\left(n\right)}e^{(\lambda_{n}-1)s/\tau }\;$$

where the tensor of weights is defined by the set of rank‐1 matrices $J_{n}=W^{\mathrm{out}}|Q_{n}\rangle \left\langle Q_{n}^{-1}\right|W^{\mathrm{in}}$.

### 4.7 lRNN and the Laplace Transform of System State

Assuming the existence of a time $t^{*}$ such that a matrix $W^{\mathrm{out}}$ maps the lRNN state into the system observable given as input $\omega \left(t\right)=W^{\mathrm{out}}r\left(t\right)$, the open‐ and closed‐loop dynamics were in principle equivalent [30, 31]. This property allowed us to link the kernel of the autoregressive model (closed‐loop dynamics) to the spectrum of the learned synaptic matrix $\tilde{W}$, and consequently, to the resulting finite Koopman approximation, as we will see in the following.

#### 4.7.1 Open‐Loop Representation

The autoregressive formula expressed in Equation (3) can be reformulated as:

(28) $$\omega_{j}\left(t\right)=\sum_{k=1}^{M}\int_{0}^{t}G_{jk}\left(t-t^{\prime }\right)\omega_{k}\left(t^{\prime }\right)dt^{\prime }+\omega_{j}\left(0\right)\;$$

The convolution theorem for the Laplace transform leads to:

(29) $$\mathrm{LT}\left\{\omega_{j}\right\}\left(\sigma \right)=\sum_{k=1}^{M}\mathrm{LT}\left\{G_{jk}\right\}\left(\sigma \right)\mathrm{LT}\left\{\omega_{k}\right\}\left(\sigma \right)+\frac{\omega_{j}\left(0\right)}{\sigma }$$

here, $\mathrm{LT}\left\{G_{jk}\right\}=\int_{0}^{\infty }G_{jk}\left(t\right)e^{-\sigma t}dt$ denotes the Laplace transform of the kernel that is

(30) $$\mathrm{LT}\left\{G_{jk}\right\}\left(\sigma \right)=\sum_{n=1}^{N}\frac{J_{jk}^{\left(n\right)}}{\sigma -\frac{\lambda_{n}-1}{\tau }}\;$$

Thus, in matrix formalism the Laplace transforms of the system observables result to be

(31) $$\mathrm{LT}\left\{\omega \right\}\left(\sigma \right)=\frac{L^{-1}\left(\sigma \right)}{\sigma }\omega \left(0\right)\;$$

where the matrix $L\left(\sigma \right)\in R^{M\times M}$ has elements $L_{ij}\left(\sigma \right)=\delta_{ij}-\mathrm{LT}\left\{G_{ij}\right\}\left(\sigma \right)$.

#### 4.7.2 Closed‐Loop Representation

The closed‐loop network evolved according to Equation (5). Assuming a diagonalizable synaptic matrix $\tilde{W}$, it can be factorized as $\tilde{W}=\tilde{Q}\tilde{\Lambda }{\tilde{Q}}^{-1}$, where $\tilde{Q}$ is a matrix whose columns are the right eigenvectors of $\tilde{W}$ and $\tilde{\Lambda }$ is a diagonal matrix containing the associated eigenvalues. The spectral decomposition of the learned synaptic matrix leads to a decoupled set of linearly evolving projections $v\left(t\right)={\tilde{Q}}^{-1}r\left(t\right)$ whose N elements evolves independently as

(32) $$v_{n}\left(t\right)=v_{n}\left(0\right)e^{({\tilde{\lambda }}_{n}-1)t/\tau }\;$$

These variables serve as a basis to decompose the system observables $\omega_{k}\left(t\right)$ as the sum of linearly evolving modes, resembling the Koopman decomposition. Indeed, given $r=\tilde{Q}v$ we can write

(33) $$\omega_{j}\left(t\right)=\sum_{n=1}^{N}\Xi_{jn}v_{n}\left(t\right)=\sum_{n=1}^{N}\Xi_{jn}v_{n}\left(0\right)e^{({\tilde{\lambda }}_{n}-1)t/\tau }\;$$

with matrix $\Xi \in R^{M\times N}$ defined as $\Xi =W^{\mathrm{out}}Q$ such that ω(t)=Ξv(t). The Laplace transform of the replicated observables is then

(34) $$\mathrm{LT}\left\{\omega_{j}\right\}\left(\sigma \right)=\sum_{n=1}^{N}\frac{\Xi_{jn}v_{n}\left(0\right)}{\sigma -\frac{\left({\tilde{\lambda }}_{n}-1\right)}{\tau }}$$

whose poles coincide with the eigenvalues of the estimated Koopman matrix.

Given the equivalence between the open and closed representations, $L_{ij}\left(\sigma \right)/\sigma$ shares the same poles, providing a link between the autoregressive modeling and the linear dynamic representation.

### 4.8 lRNNs and the Spectrum of the Koopman Operator

The closed‐loop representation provided a decomposition of the signal as linear evolving modes. We could explicitly express the functional form of the basis starting from open loop dynamics. By applying ${\tilde{Q}}^{-1}$ to both hand sides of Equation (2) yields:

(35) $$\begin{array}{c} v\left(t\right)=\sum_{k=1}^{M}\int_{0}^{\infty }\omega_{k}\left(t-s\right){\tilde{Q}}^{-1}e^{(W-I)s/\tau }|W_{k}^{\mathrm{in}}\rangle ds\; \end{array}$$

By resorting to the spectral decomposition in Equation (26), the above eigenmode dynamics reduces to:

(36) $$\begin{array}{ccc} v_{j}\left(t\right) & = & \sum_{k=1}^{M}\sum_{n=1}^{N}{\left[{\tilde{Q}}^{-1}|Q_{n}\rangle \left\langle Q_{n}^{-1}\right|W^{\mathrm{in}}\right]}_{jk}\\ & & \int_{0}^{\infty }\omega_{k}\left(t-s\right)e^{(\lambda_{n}-1)s/\tau }ds\; \end{array}$$

We could then expand in Taylor series the observables: $\omega_{k}\left(t-s\right)=\sum_{r=0}^{\infty }\frac{d^{r}\omega_{k}\left(t\right)}{dt^{r}}{\left(-1\right)}^{r}s^{r}/r!$. This allowed to solve the above integral leading to a functional description of the estimated eigenmodes

(37) $$\begin{array}{c} v_{j}\left(t\right)=\psi_{j}\left[\omega \left(t\right),\dot{\omega }\left(t\right),..\right]=\sum_{n=1}^{N}\sum_{k=1}^{M}A_{jk}^{\left(n\right)}\sum_{r=0}^{\infty }\frac{d^{r}\omega_{k}\left(t\right)}{dt^{r}}{\left(\frac{\tau }{\lambda_{n}-1}\right)}^{r}\; \end{array}$$

The vector of all the derivatives $d^{r}\omega_{k}\left(t\right)/dt^{r}$ fully determine the state of the observed system [33] and $\psi_{j}$ is the eigenfunction of the approximated Koopman operator depending on the full state of the system. The corresponding eigenvalue $\sigma_{j}=\left({\tilde{\lambda }}_{j}-1\right)/\tau$, and $A_{jk}^{\left(n\right)}$ is a tensor of complex weights defined by the rank‐1 matrices $A_{n}=\frac{\tau }{1-\lambda_{n}}\;{\tilde{Q}}^{-1}|Q_{n}\rangle \left\langle Q_{n}^{-1}\right|W^{\mathrm{in}}$.

### 4.9 Stochastic lRNN

The deterministic neural network described can be extended to the stochastic case, where a white noise input stimulates the reservoir, maintaining it out of equilibrium as a continuous source of new energy. Each unit receives a total input defined by the equation:

(38) $$h_{j}\left(t\right)=\sum_{k=1}^{N}W_{jk}r_{k}\left(t\right)+h_{j}^{\mathrm{in}}\left(t\right)+\eta_{j}\left(t\right)$$

where $\eta_{j}\left(t\right)$ represents an Ornstein‐Uhlenbeck process (colored noise) with a relatively small correlation time such that for the purpose of this work it could be considered as memory‐less: $\left\langle \eta_{i}\left(t\right)\eta_{j}\left(t^{\prime }\right)\right\rangle =\gamma^{2}\delta_{ij}\delta \left(t-t^{\prime }\right)$. An optimal standard deviation γ can be identified to maximize the accuracy of the linear map $W^{\mathrm{out}}$.

In the closed‐loop formulation, the input can be expressed as:

(39) $$h_{j}\left(t\right)=\sum_{k=1}^{N}{\tilde{W}}_{jk}r_{k}\left(t\right)+\eta_{j}\left(t\right)$$

Consequently, the associated Langevin equation for the reservoir is given by:

(40) $$\tau dr=-rdt+\tilde{W}rdt+d\eta \;.$$

In this context, the introduced autoregressive description and linear decomposition remain valid when considering the expected values of the system.

### 4.10 Functional Connectivity and Functional Connectivity Dynamics

Functional Connectivity (FC) is a measure of the degree of co‐activation over time of different brain regions. Although FC does not allow to infer the directional flow of information among the nodes of the brain network, it has proven to provide valuable insights into the functional organization of the central nervous system [85]. Mathematically, the FC between two brain regions labeled A and B, is usually measured as the correlation function

(41) $${\mathrm{FC}}_{AB}\left(t;T\right)=\mathrm{cor}\left[X_{A}\left(t;T\right),X_{B}\left(t;T\right)\right]\;$$

where cor is the Pearson correlation coefficient, and $X_{A}\left(t;T\right)$ and $X_{B}\left(t;T\right)$ are the BOLD signals from region A and B, respectively, in the time window [t,t+T].

Temporal changes of functional connectivity were evaluated by computing the Functional Connectivity Dynamics (FCD) [86, 87]. FCD was defined as the correlation between vectorized FC matrices evaluated in time‐shifted chunks of BOLD activity:

(42) $${\mathrm{FCD}}_{A,B}\left(\delta t,t;T\right)=\mathrm{ucor}\left[{\mathrm{FC}}_{A}\left(t;T\right),{\mathrm{FC}}_{B}\left(t+\delta t;T\right)\right]\;$$

where δt is a constant time shift, and ucor is the correlation between the vectors composed of the upper‐diagonal elements of the two FC matrices involved.

Throughout the whole paper FC was computed with t=0 s and T=387.5 s, while FCD uses T=75 s time shift δt multiples of 12.5 s.

### 4.11 Functional Connectivity in lRNN

Once a lRNN capable to reproduce the systems observables $\omega_{j}\left(t\right)$ was available, the elements of the FC matrix could be inferred directly from the lRNN parameters. The computed Functional Connectivity of the simulated signals depends on the covariance matrix estimated from time t over a time window of length T. Considering large time windows T and assuming a zero time average ${\left\langle \omega_{j}\left(t\right)\right\rangle }_{t}≃0$, it holds:

(43) $$\begin{array}{ccc} {\mathrm{cov}}_{ij}\left(t;T\right) & = & \int_{t}^{t+T}\omega_{i}^{*}\left(t^{\prime }\right)\omega_{j}\left(t^{\prime }\right)dt^{\prime }\\ & = & \sum_{r,s}\Xi_{ir}^{*}\Xi_{js}\int_{t}^{t+T}v_{r}^{*}\left(t^{\prime }\right)v_{s}\left(t^{\prime }\right)dt^{\prime }\\ & = & \sum_{r,s}\Xi_{ir}^{*}\Xi_{js}\Omega_{rs}\left(t;T\right) \end{array}$$

Here, we are making use of the spectral decomposition in Equation (7) and we define $\Omega_{rs}\left(t;T\right)$ as the estimated covariance between the OU modes $v_{r}$, $v_{s}$. From the simulations, it results that only a few slow uncorrelated modes had a significant weight Ξ in the decomposition. In this scenario, the covariance matrix was well approximated by the dot product between the representational vectors in the functional space:

(44) $${\mathrm{cov}}_{ij}\left(t;T\right)\approx \Xi_{i}\cdot \Xi_{j}$$

Consequently for the correlation matrix it holds

(45) $${\mathrm{FC}}_{ij}\left(t;T\right)\approx S_{C}\left(\Xi_{i},\Xi_{j}\right)$$

where $S_{C}$ indicates the cosine similarity function, i.e., the Pearson correlation between row and column vectors composing $\Xi^{*}$ and Ξ, respectively, under the assumption of a zero mean for the system observables.

### 4.12 Simulation Parameters

Network parameters had been selected through grid search methods to optimize prediction accuracy. The reservoir state was initialized in a zero‐activity state and was disrupted from equilibrium by the input for a transient period of 20s before learning. This period corresponds to the $t^{*}$ mentioned in the preceding paragraphs. The input signal was linearly interpolated, and the system's dynamics were resolved using the Euler–Maruyama method with a time step that was one‐hundredth of the sampling interval. The spectral radius ρ of the synaptic matrix, used for fMRI analysis, was set to 0.5, and the time constant τ was 1.0. The standard deviation $g^{\mathrm{in}}$ of the input matrix $W^{\mathrm{in}}$ was set to 1. The thermal noise shown in Figure 4 had a regularizing effect. Consequently, we did not employ further regularization for the readout regression. For the example system depicted in Figure 1, the parameters were ρ=0.5,τ=0.1 with no regularizers, and $g^{\mathrm{in}}$ equal to the inverse of the square root of the number of inputs. The stochastic scenario was characterized by thermal noise with a standard deviation of $10^{-5}$ and ridge regression with a regularization of $10^{-7}$. For cases with missing variables, the parameters were set to ρ=0.6,τ=0.25.

Hankel DMD analyzes in Figure 3 were all performed choosing a truncation rank r preserving 0.99999 of the energy in the computed SVD and memory length q=5.

### 4.13 Clustering Analysis

The clustering analysis was performed on the resulting spectrum of the data by discretizing the complex space into a grid with a real resolution of $0.00025s^{-1}$, and an imaginary resolution of $0.01s^{-1}$.

To further reduce the dimensionality of the data and get an interpretable 3D representation, Linear Discriminant Analysis (LDA) was employed. LDA was a widely used technique in machine learning [80]. It was a supervized learning method that aims to reduce the dimensionality of the data while preserving the discriminatory information between different classes. The core idea was to find a linear transformation that maximizes the ratio of the between‐class scatter $S_{b}$ to the within‐class scatter $S_{w}$. This optimization process equals to solve the following generalized eigenvalue problem

(46) $$S_{b}l=\lambda S_{w}l$$

The optimal projection matrix L to a subspace of dimension k was given by the eigenvectors l corresponding to the largest k eigenvalues.

### 4.14 Linear Decoders

To identify frequency patterns that discriminate brain areas in the discretized frequency‐vector representation, we trained a multi‐class linear decoder that maps each frequency vector to a one‐hot target indicating the corresponding area. Let M=11 denote the number of areas and D the dimensionality of the discretized frequency representation. Pooling all subjects and all 100 reservoir realizations, we arranged the data into the feature matrix

$$F=\left[f_{1},\cdots ,f_{S}\right]\in R^{D\times S}$$

where each column $f_{n}\in R^{D}$ is one sample and S is the total number of samples (i.e., 20×100=2,000). We encoded area labels in the one‐hot matrix

$$Y=\left[y_{1},\cdots ,y_{S}\right]\in {\left\{0,1\right\}}^{M\times S}$$

where $y_{n}\in {\left\{0,1\right\}}^{M}$ has a single 1 at index corresponding the area of sample n, and zeros elsewhere. We estimated the decoding matrix $W\in R^{M\times D}$ by solving the matrix least‐squares problem

$$W=\arg\min_{W_{0}}\;{∥Y-W_{0}F∥}_{F}^{2}$$

The minimum‐norm solution is given by $W=YF^{+}$, where $F^{+}$ denotes the Moore‐Penrose pseudoinverse. The k‐th row $W_{k:}$ defines the linear decoder for area k. The output of the area‐k decoder for a single sample $f_{n}$ is the scalar

$${\hat{y}}_{k}\left(n\right)=\sum_{j=1}^{D}W_{kj}\;F_{jn}$$

Per‐area accuracy for area k was defined as the fraction of samples with true label k for which ${\hat{y}}_{k}\left(n\right)$ attains the maximum across areas.

### 4.15 Manuscript Language Optimization

To enhance the clarity, coherence and readability of the manuscript, we employed large language models (LLMs) for language editing and refinement. All edits assisted by the model were subsequently reviewed by us to ensure accuracy and to preserve the intended scientific meaning.

### 4.16 Code Availability

All codes are available at https://github.com/gdianto/LinearRNNforFMRIdigitaltwins

## Conflicts Of Interest

The authors declare no conflicts of interest.

## Supporting information


**Supporting File**: advs74692‐sup‐0001‐SuppMat.pdf.

## Data Availability

The data that support the findings of this study are available on request from the corresponding author. The data are not publicly available due to privacy or ethical restrictions.
