# Supplementary material for: Linearizing and Forecasting: A Reservoir Computing Route to Digital Twins of the Brain
Source: Adv Sci (Weinh). 2026 Mar 19;13(28):e17234. doi: 10.1002/advs.202517234 (PMC13185859; doi:10.1002/advs.202517234)
Supplement: Supplementary file 1 — Supporting File: advs74692‐sup‐0001‐SuppMat.pdf. [file ADVS-13-e17234-s001.pdf]

SUPPORTING INFORMATION

Linearizing and forecasting: a reservoir computing route to digital twins of the brain

Gabriele Di Antonio[1,2,3], Tommaso Gili[4], Andrea Gabrielli[1,2], and Maurizio Mattia[3,*]

[1] *"Enrico Fermi" Research Center - CREF, 00184 Rome, Italy*
[2] *Dip. di Ingegneria Civile, Informatica e delle Tecnologie Aeronautiche, Università degli Studi "Roma Tre", 00146 Rome, Italy*
[3] *Natl. Center for Radiation Protection and Computational Physics, Istituto Superiore di Sanità, 00161 Rome, Italy*
[4] *Networks Unit, IMT Scuola Alti Studi Lucca, 55100 Lucca, Italy*
[*] *Corresponding author: maurizio.mattia@iss.it*

February 6, 2026

Supplementary Figures

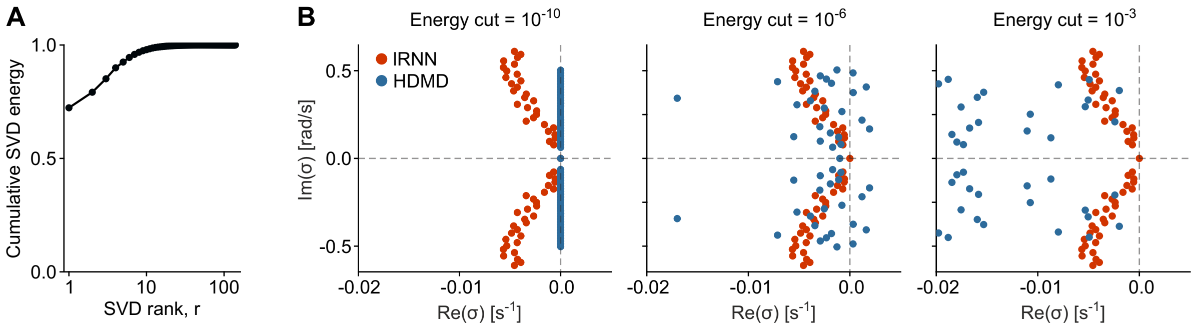

Supplementary Figure 1: Pole spectra inferred by lRNN and by Hankel-DMD. A) Cumulative energy captured by the singular-value decomposition (SVD) as a function of truncation rank for the representative subject 5 from the rs-fMRI dataset used in this study. B) Pole spectra inferred by lRNN (linear recurrent neural networks, red) and by Hankel-DMD (demixed mode decomposition, blue). See main text for details. In each panel, the same lRNN pole spectrum is shown alongside a Hankel-DMD spectrum computed using a different SVD truncation rank. The energy cutoff associated with the chosen truncation rank decreases from left to right.
